# Hepatitis B virus vaccination status of sixth year medical students

**DOI:** 10.1186/1471-2334-13-S1-P29

**Published:** 2013-12-16

**Authors:** Oana Streinu-Cercel, Anca Streinu-Cercel, Liliana Lucia Preoțescu, Adrian Streinu-Cercel

**Affiliations:** 1 Carol Davila University of Medicine and Pharmacy, Bucharest, Romania; 2 National Institute for Infectious Diseases “Prof. Dr. Matei Balş”, Bucharest, Romania

## Background

Hepatitis B virus (HBV) infection can associate a high risk of progression to chronic hepatitis, particularly when infection occurs at younger ages. For this particular reason, newborn vaccination has been introduced in national immunization schedules in countries where the infection has a medium to high prevalence.

We assessed the vaccination status of students in their final year of medical university training during 2011-2013. This category is at risk of professional exposure to HBV and given the timeline of introduction of HBV vaccination in Romania, they probably have not been vaccinated at birth, but rather during middle or high school education.

## Methods

We informed all medical students attending the Infectious Diseases rotation in their 6^th^ year of medical school regarding the possibility of determining their HBV serological status and filling out a standardized questionnaire. We correlated this information with the results of lab tests. We analyzed data with SPSS Statistics 20 (IBM).

## Results

A total of 59 students (of which 17 were males) filled out the questionnaire. The mean age was 25±3 years. Of these students, 40 were aware of their vaccination status for HBV but only 10 were able to mention the exact date of the first vaccination and 13 were able to mention their last booster shot. In the group of 47 students that had recent lab results, all had negative HBs antigen, and the median HBs antibody (Ab) lab result was: 134±497. Of them, 11 were considered as unprotected (HBsAb≤10), 12 had values between 10-100, 15 between 100-1000 and 9 above 1000.

## Conclusion

Most of the students that participated in this study were aware of their vaccination status for HBV, however, many of them were considered as unprotected, based on HBsAb results. Cellular immunity is also known to play an important role in protection from HBV infection; however, at this point no instruments are available to quantify specific memory cells in current clinical practice.

